# Quantification of head leakage radiation in CyberKnife robotic radiosurgery systems using a multimodal approach

**DOI:** 10.1038/s41598-025-18689-1

**Published:** 2025-10-07

**Authors:** Sandeep Singh, Manindra Bhushan, Pawan Kumar Singh, Debasish Sahoo, Supratik Sen, Dipesh, Abhay Kumar Singh, Mahipal, Munish Gairola

**Affiliations:** 1 Department of Radiation Oncology, Division of Medical Physics, Rajiv Gandhi Cancer Institute and Research Center, New Delhi, India; 2 Department of Radiation Oncology, VMMC & Safdarjung Hospital, New Delhi, India; 3 Department of Radiation Oncology, Homi Bhabha Cancer Hospital and research Center, Visakhapatnam, India; 4 Department of Physics, GLA University, Mathura, Uttar Pradesh India

**Keywords:** Leakage, In-patient plane, Out of patient plane, OSLD, Survey meter, Applied physics, Health care

## Abstract

This study aims to measure the head leakage radiation of a CyberKnife system (Model-S7 (without MLC)) in-patient and out-of-patient planes using optically stimulated luminescence dosimeters (OSLDs), vented ionization chambers, and a pressurized ionization chamber-based survey meter, and to compare the measured leakage levels with the limits specified by the International Electrotechnical Commission (IEC) standards. In this study, a CyberKnife LINAC equipped with a 6 MV flattening filter-free (FFF) beam and a maximum circular field size of 6 cm in diameter was utilized. Leakage radiation was assessed for both IRIS and FIXED collimators in their fully closed positions. Three independent measurement techniques were employed to quantify leakage: optically stimulated luminescence dosimeters (OSLDs), vented ionization chambers (ICs), and a pressurized ionization chamber–based survey meter (SM). Leakage measurement points were taken as per the IEC 60601-2-1 report both for in-patient plane and out-of-patient plane. The total number of monitor units (MU) delivered were 500 MU’s. The measured average in-patient plane leakage was 0.015% using IC, 0.100% using OSLD and 0.010% using SM, that is well within the tolerance limit of 0.1% according to IEC guidelines. The average leakage for out of patient plane was 0.037%, which is well within the limit of 0.5% as per IEC guidelines. The study measured head leakage radiation of the CyberKnife System using OSLD, ionization chambers, and a survey meter. Leakage levels for both in-patient and out-of-patient planes were significantly below the IEC 60601-2-1 standard limits. This confirms the system’s compliance with safety guidelines, ensuring minimal radiation exposure.

**Supplementary Information:**

The online version contains supplementary material available at 10.1038/s41598-025-18689-1.

## Introduction

The stringent regulation and oversight of radiation delivering units by the Atomic Energy Regulatory Board, Mumbai, are fundamental for ensuring the safe and effective application of radiation treatment in India^1^. Before any radiation delivering unit can be used for clinical purposes, it must undergo a comprehensive evaluation process to meet rigorous national and international standards^2,3^. This process culminates in the issuance of a type approval certificate and affirming compliance with stringent criteria encompassing electrical, mechanical, dosimetric, and radiation safety parameters. An essential component of radiation safety regulations is the control of leakage radiation that may pose risks to both patients and healthcare personnel. Leakage radiation refers specifically to the unintended radiation that escapes from the treatment head in directions other than the intended beam path, arising through mechanisms such as transmission through or around secondary collimators (jaws or MLCs), scatter from internal head components, leakage through shielding imperfections, and minimal transmission through gantry or support structures. It is distinct from the primary beam and from scattered radiation within the treatment room, which result from the interaction of the primary beam with the patient or surrounding surfaces. To address this, organizations like the IEC and national bodies set strict limits and guidelines to protect people outside the treatment area and ensure accurate radiation delivery^4,5^. Quality Assurance (QA) protocols are crucial in this process, involving thorough testing of equipment to ensure effective shielding and collimation^6,7^. By following these protocols, radiotherapy facilities improve treatment accuracy and maintain a safe environment for patients and staff. Measuring head-leakage radiation in clinical linear accelerators is crucial for ensuring the safety of patients, radiation workers, hospital staff, and the public. When the treatment beam is active, the radiation within the therapy room includes primary, scattered, and head-leakage photons. The effectiveness of shielding and head design plays a crucial role in determining leakage levels, making it necessary to measure and verify specific leakage values for each accelerator model after installation at each site^8^.

The CyberKnife system, a robotic radiosurgery platform developed by Accuray Inc., differs substantially from conventional linear accelerators in its delivery geometry and collimator configuration, presenting unique challenges in shielding design. The literature on CyberKnife shielding requirements emphasizes its distinctive leakage profile due to the fixed circular, IRIS, and MLC collimators, as well as the non-isocentric, multi-angle delivery approach. Several published studies and technical reports, including those by AAPM Task Group 135^9^, and Kry et al.^10^ and Kank et al.^11^ have discussed the shielding implications for CyberKnife systems. These documents highlight that room shielding and head leakage considerations must account for the absence of a flattening filter, frequent beam-on transitions, and close proximity of leakage pathways to the treatment head due to its compact design and robotic mobility.

The head design of the CyberKnife system has undergone significant evolution across its various models, leading to notable improvements in radiation shielding and leakage control. Early-generation models such as the G3 and G4 incorporated basic tungsten-based shielding with fixed circular collimators, resulting in relatively higher leakage levels, often approaching the upper limit of IEC 60601-2-1 tolerances (~ 0.2% of the primary beam). The introduction of the VSI model brought the Iris variable aperture collimator, prompting modifications in internal beam path design and shielding architecture. These enhancements led to modest reductions in off-axis leakage. A major advancement occurred with the M6 model, which integrated a high-resolution multileaf collimator (InCise™ MLC). This necessitated a substantial redesign of the head structure, incorporating improved beam-line shielding, better leaf-end and interleaf attenuation, and more efficient scatter management. The most recent model, CyberKnife S7, features a fully optimized head design with refined shielding geometry developed through advanced Monte Carlo simulations. It incorporates low-leakage alloy materials, precise collimator encapsulation, and improved containment of scatter radiation. As a result, the S7 demonstrates the lowest head leakage among all models, with values consistently below 0.05% of the primary beam, well within international regulatory limits. These iterative advancements underscore the manufacturer’s ongoing efforts to enhance radiation safety without compromising treatment precision.

The primary aim of this study is to comprehensively evaluate the head leakage radiation emitted from the CyberKnife System, both within the patient treatment plane (in-patient plane) and outside of it (out-of-patient plane) utilizing three types of dosimetric devices, vented type ionization Chambers, pressurized ionization chamber-based survey meter and OSLD’s. The assessment focuses on determining whether the leakage levels comply with the safety thresholds established by the International Electrotechnical Commission (IEC) standard 60601-2-1^4^, which sets global guidelines for radiation safety in medical linear accelerators (LINAC).

## Methods and materials

**Fig. 1:**
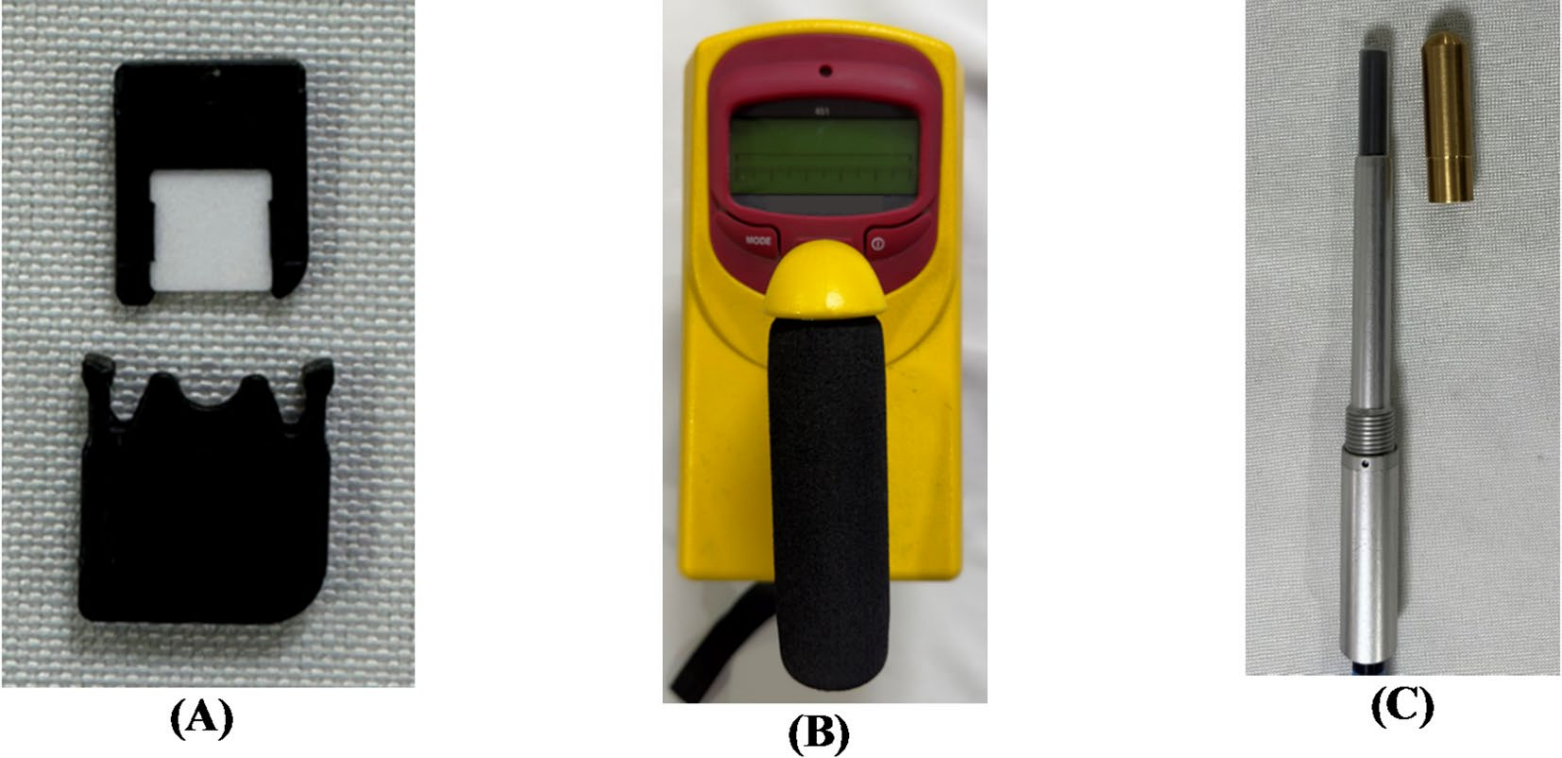
The dosimetric devices used in the study (A) Optically Luminescence Dosimeter, (B) Pressurized ion chamber-based survey meter, (C) Farmer type ion chamber with brass build-up cap. High-accuracy, real-time dose measurements can be obtained using ionization chambers. Complementing these, survey meters offer instantaneous dose rate readings, enabling quick assessments and continuous radiation monitoring. In contrast, OSLDs allow for simultaneous assessment of leakage at multiple locations in a single exposure, significantly reducing the time required for comprehensive leakage evaluations.

Medical linear accelerators undergo rigorous scrutiny during acceptance and commissioning. One of criteria is measurement and control of in-patient plane leakage. Understanding, measuring, and controlling this leakage is vital for several reasons, each contributing to the overarching goal of ensuring patient safety, treatment efficacy, and compliance with regulatory standards. The primary concern with in-patient plane leakage is its potential to deliver unintended doses of radiation to healthy tissues and organs, adjacent to the treatment area^12,13^. Even small doses of unintended radiation can lead to long-term side effects, such as secondary cancers^14–16^. Therefore, monitoring and minimizing leakage is crucial. Accurate measurement of in-patient plane leakage involves using sophisticated equipment such as ionization chambers, film dosimeters, and optically stimulated luminescence dosimeters (OSLDs). These tools are essential for detecting and quantifying the leakage radiation. We used three dosimetry tools to measure the leakage radiation in the CyberKnife machine shown in Fig. 1.

**Fig. 2:**
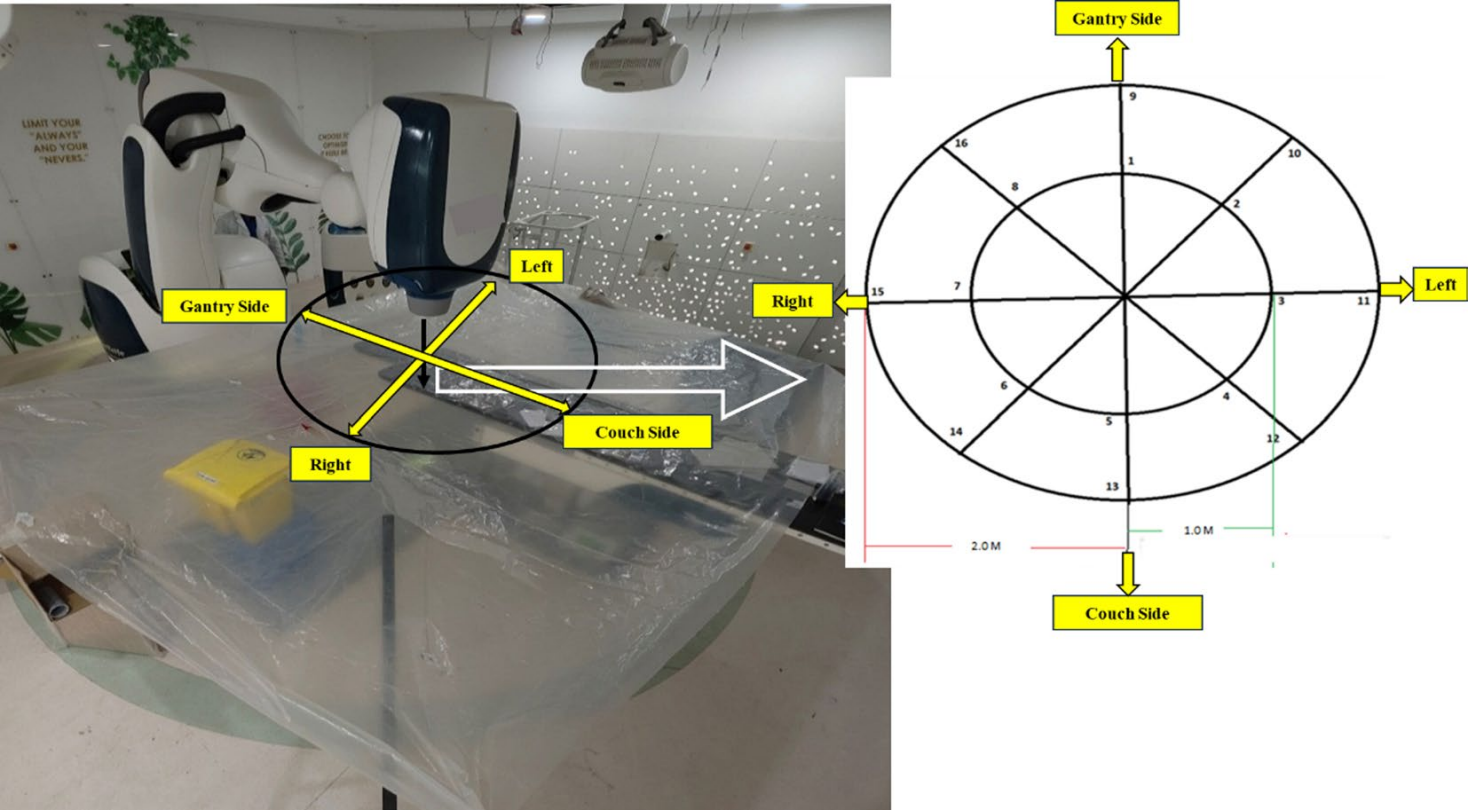
Schematic representation of the radiation leakage measurement setup performed as per IEC guidelines. The left panel depicts the actual clinical arrangement, where a plastic sheet was placed on the treatment couch to position dosimeters for simultaneous measurement at designated points. Directional markers—Gantry Side, Couch Side, Left, and Right—are indicated to define the spatial orientation. The right panel illustrates the accessible 16 measurement points arranged along two radial distances (1 m and 2 m) from the isocenter.

**Fig. 3:**
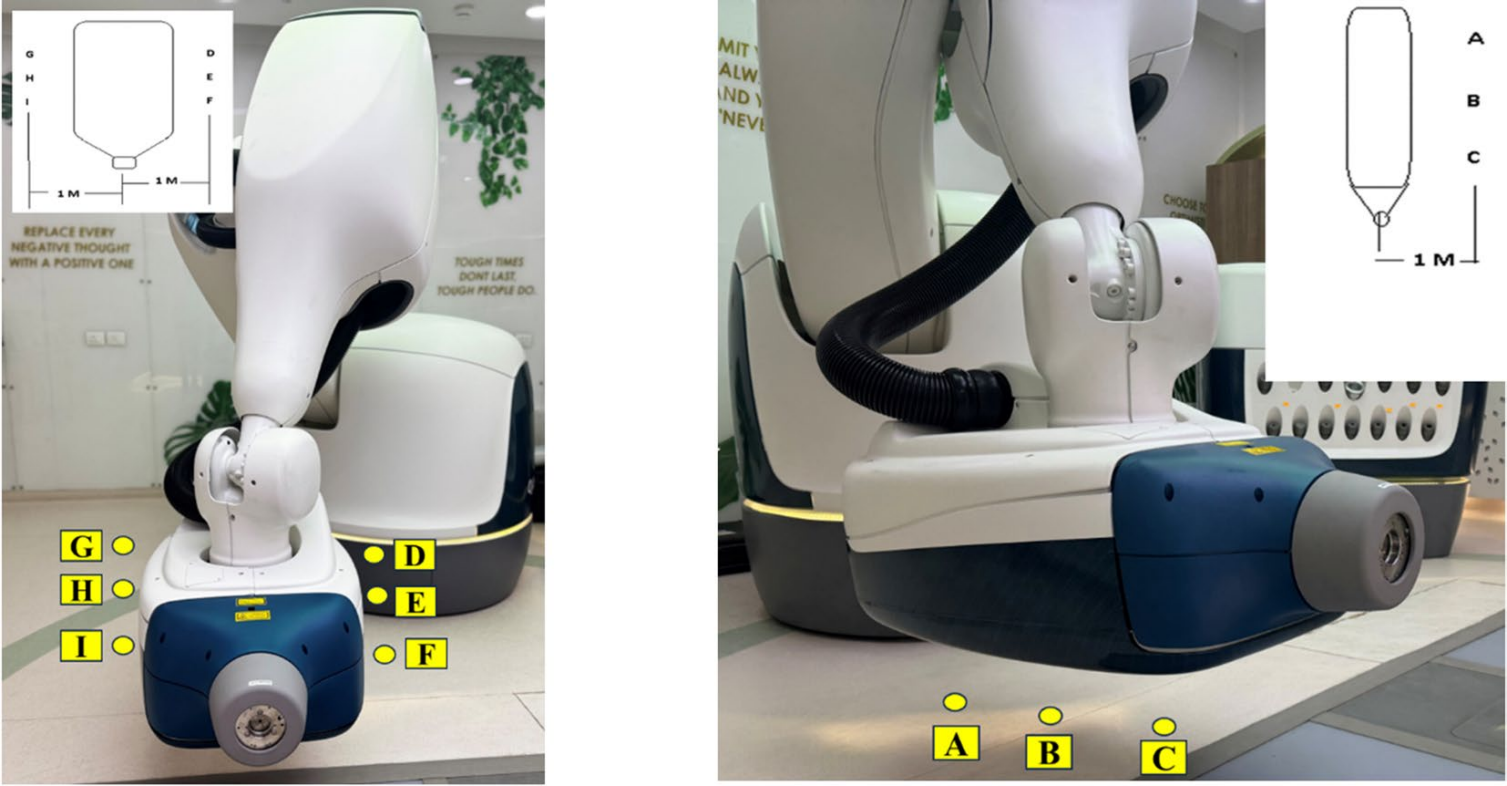
CyberKnife S7 system showing detector positions for out-of-patient plane leakage measurement around the linac head. **(left)**: Front and lateral view indicating measurement points D, E, F (right lateral), and G, H, I (left lateral) at 1 m from the linac head surface in the transverse plane. **(right)**: Inferior view showing measurement points A, B, and C located vertically below the linac head at a 1-meter distance along the beam axis. Insets show schematic diagrams for respective detector positions.

The Farmer-type ionization chamber (FIC) plays a pivotal role in radiotherapy, and is the gold standard for measurement^17,18^. In this study we had used PTW30013 FC-65 0.6 cm^3^ (PTW GmBH, Freiberg Germany) FIC with brass build-up cap, placed on 24 points outlined by IEC-60601-2-1. 16 are accessible and locations are shown in Fig. 2. The FIC was also used for the out-of-patient plan measurements and the points are shown in Fig. 3. The survey meter (Fluke 451P Radiation Detector), equipped with a 230-cc volume pressurized air ionization chamber, maintains accuracy within 10% of the reading between 10% and 100% of the full-scale indication on any range, exclusive of energy response. This detector can detect beta radiation above 1 MeV, as well as gamma and x-rays above 25 keV^19^. The OSLD system (RadPro International GmbH, Germany) comprises multiple myOSLchips, a handheld manual OSL reader, an eraser including 24 blue power light-emitting diodes for annealing, and software for registration, calibration, and measurement^20^. The myOSLchips had a disk of dimension 4.7 × 4.7 × 0.5 mm, of Beryllium Oxide (BeO). The size of dosimeter with plastic holder is 10 × 9.5 × 2 mm. It has the energy response from 16 KeV to 25 MeV. The dose range of the chip is 0.05 mGy to 10 Gy and dose response is linear up to10 Gy.

OSLD dose uncertainty was estimated using the standard deviation from three independent readouts per chip after calibration and background correction. Each OSLD was calibrated using a known dose under standard beam conditions to determine its individual sensitivity factor. Background signals measured post-annealing were subtracted from the final readings to eliminate residual luminescence. These values are all at or above the minimum detection threshold of 0.05 mGy (with some outliers), ensuring measurement reliability.

The dose comparison in this study was performed at d_max_ (dose at maximum depth), 6 cm diameter field size at the central axis at Nominal Treatment Distance (NTD) and represented as ‘Percentage Dose fraction’. According to the International Electrotechnical Commission (IEC) 60601-2-1 standard, tolerance for maximum permissible photon head leakage is 0.2%, with an average value of 0.1%, of the primary beam, measured along the central axis^4^. The formula used to calculate the Relative Dose was given as,

$${\text{Relative Dose}}(\% )=\frac{{{\text{Dose at point of interest}}}}{{{\text{Dose at the reference point on the central axis}}}} \times 100$$

The “Dose at point of interest” represents the dose measured at the point of interest with a closed collimator (0 cm² field size), and “ dose at the reference point on the central axis” is the reference dose on the central axis using a 6 cm diameter cone.

All head leakage measurements were performed under closed-collimator conditions, using the smallest possible aperture setting for both IRIS and FIXED collimators, corresponding to an effective field size of 0 cm² (Fully closed setting in IRIS and shielded cone in FIXED collimator). No additional phantom or scattering object was placed in the beam path. This configuration ensured that there was no contribution from the primary beam or patient-scattered radiation. The measurements thus captured leakage radiation originating solely from within the treatment head and surrounding shielding structures. These conditions were selected in alignment with IEC recommendations for head leakage testing, where the goal is to isolate and quantify stray radiation emitted from the accelerator head in the absence of beam-target interaction or phantom scatter.

## Results

### In-patient plane leakage values

**Fig. 4:**
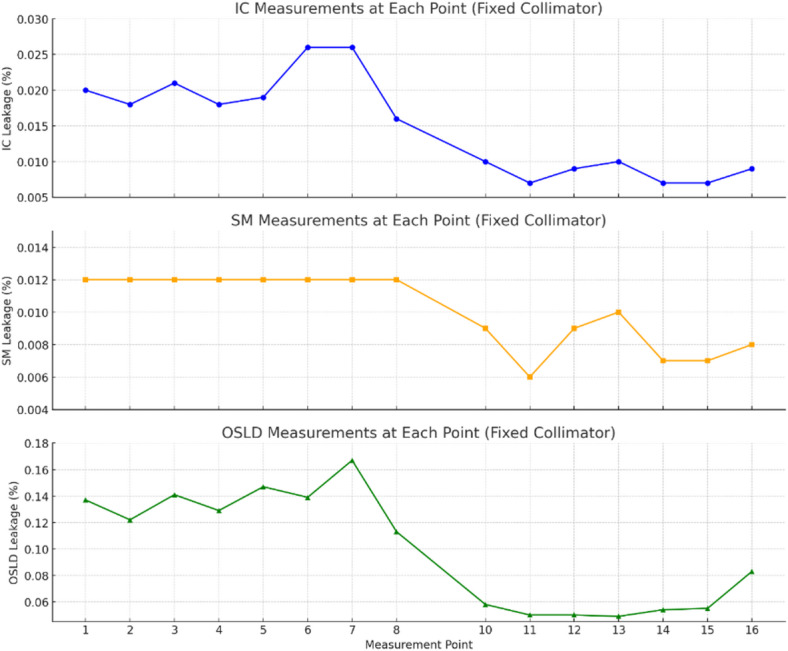
Shows the in-patient plane leakage radiation measured using three different dosimetric tools for the fixed collimator configuration. (Top): Ionization Chamber (IC) measurements show high sensitivity and precision, capturing point-to-point leakage variation in the range of 0.005–0.030%. (Middle): Survey Meter (SM) readings display consistent but slightly lower values due to its large volume and slower response, with leakage ranging from 0.004–0.015%. (Bottom): Optically Stimulated Luminescence Dosimeter (OSLD) measurements indicate higher values (0.045–0.18%), likely due to accumulated sensitivity and background signal, providing full-field spatial coverage in a single exposure.

**Fig. 5:**
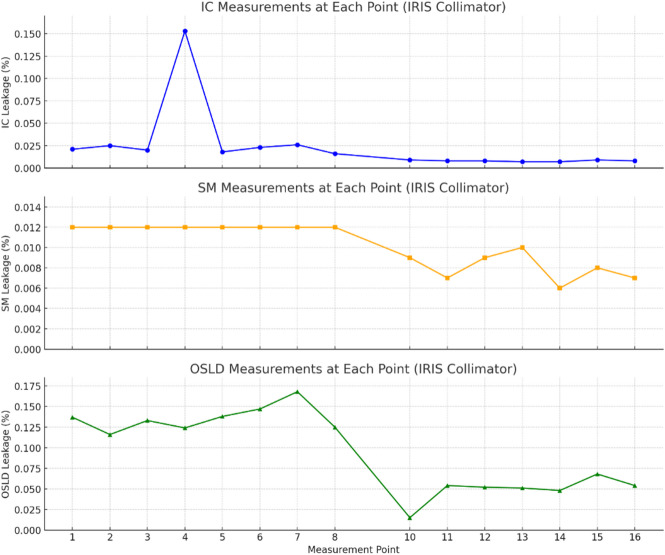
Shows the in-patient plane leakage radiation measurements for the IRIS collimator using three dosimetric methods—Ionization Chamber (IC), Survey Meter (SM), and Optically Stimulated Luminescence Dosimeter (OSLD). The IC plot (top) shows point-specific variations with a peak at point 4 and values up to 0.153%. The SM plot (middle) indicates relatively uniform readings around 0.012%. The OSLD plot (bottom) displays broader variation and higher leakage detection, peaking at 0.168% (point 7. This figure highlights the spatial distribution of leakage and the relative sensitivity of each dosimetric tool.

The Ionization Chamber measurements for the IRIS setup had a maximum radiation leakage of 0.1741% and an average leakage of 0.0253%, whereas the FIXED Collimator had a maximum leakage of 0.0264% and an average of 0.0148%. The Survey Meter results showed a maximum leakage of 0.012% and an average of 0.011% for the IRIS, and a maximum of 0.012% and an average of 0.010% for the FIXED Collimator. OSLD measurements for the IRIS setup had a maximum leakage of 0.168% and an average leakage of 0.095%, while the FIXED Collimator had a maximum of 0.167% and an average of 0.100%. The radiation leakage is significantly below the permissible limits of 0.2% for maximum leakage and 0.1% for average leakage, based on the maximum absorbed dose at the nominal treatment distance (NTD) for a 6 cm radiation field size as given by IEC standards. Figures 4 and 5 shows the different values of leakage for all the points using IRIS and FIXED collimator.

Although a perfectly symmetric collimator design—whether fixed or IRIS—would theoretically produce similar leakage levels across symmetric measurement points (e.g., points 1–8 and 9–15), resulting in an idealized Heaviside-like distribution, practical deviations are commonly observed. These arise due to small mechanical asymmetries in the collimator structure, non-uniform shielding arrangements within the treatment head, and subtle variations in internal component positioning. Additionally, differences in room scatter conditions and minor detector placement deviations can contribute to point-to-point variation. In the case of the IRIS collimator, a distinct peak in leakage was observed at point 4, which was not mirrored at the corresponding radial position (e.g., point 12). This asymmetry likely stems from the internal mechanical complexity of the IRIS collimator, which employs a dynamic aperture adjustment mechanism that may not provide uniform shielding across all orientations. Furthermore, the CyberKnife system’s non-isocentric beam delivery and robotic arm geometry can introduce angular-specific scattering and leakage paths. As a result, the measured leakage distribution deviates from ideal symmetry and highlights the influence of both design and setup factors on real-world radiation leakage patterns. (In Supplementary file Table: S1 and S2 shows in-patient plane leakage for FIXED and IRIS collimators, measured with ion chamber (IC), survey meter (SM), and optically stimulated luminescent dosimeters (OSLDs)).

### Out of patient plane leakage values

**Fig. 6:**
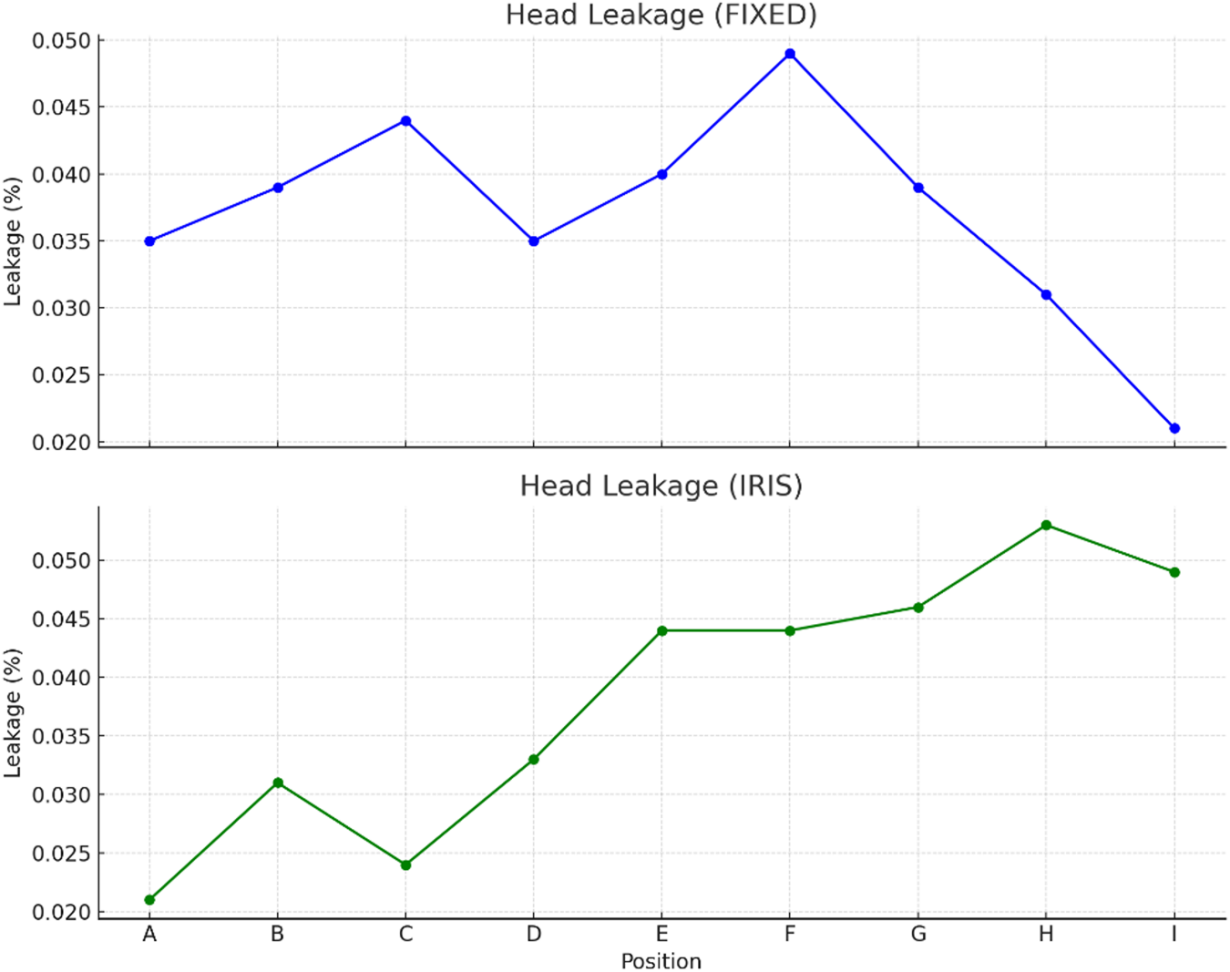
Head leakage measurements for FIXED and IRIS collimators at various positions. The graphs present head leakage (%) recorded at measurement positions A through I for two collimator configurations: FIXED (top) and IRIS (bottom). Leakage values were obtained using identical measurement conditions and normalized to the reference beam output. The FIXED collimator shows relatively consistent leakage across positions, peaking at position F. In contrast, the IRIS collimator demonstrates an increasing trend in leakage from positions A to H, with the highest leakage observed at position H. These results help quantify head leakage characteristics for different collimator systems used in stereotactic radiosurgery.

The ionization chamber was the only device used for out-of-patient treatment leakage measurements, as it offers superior sensitivity, stability, and real-time readout—qualities essential for accurately detecting the very low leakage doses encountered in these regions. In contrast, OSLDs, while advantageous for simultaneous multi-point measurements, exhibit limited sensitivity at very low dose levels and greater variability, particularly when the accumulated dose is minimal. Likewise, the survey meter is more suitable for rapid qualitative checks and dose rate assessments but lacks the precision required for detailed, point-by-point quantitative analysis of low leakage levels. Additionally, practical considerations such as spatial accessibility and the need for stable detector positioning around the treatment room further justified the exclusive use of the ionization chamber in these measurements. When measured at a distance of one meter from the source path, the highest observed radiation leakage was 0.053% for the IRIS collimator and 0.049% for the FIXED collimator. (In Supplementary file Table S3: presents Other than Patient Plane Leakage for both fixed and IRIS collimator) (Fig. 6)

## Discussion

### Comparison with similar studies and dosimetry methods

Our measurements revealed notable differences between the dosimetric techniques: optically stimulated luminescence dosimeters (OSLDs) registered in-patient plane higher than the ionization chamber (IC) and survey meter readings, even though all fell well below regulatory limits (≤ 0.1%). This disparity is consistent with characteristics reported in the literature. For example, Jaradat and Biggs measured linac head leakage using both a large-volume ion chamber (100 cm^3^) and OSL dosimeters, finding that leakage was generally under 0.1% of the primary beam at 1 m^21^. Their use of a 100 cm^3^ chamber was necessitated by the extremely low signal from leakage radiation, underscoring the challenge of measuring such small doses.

In our study, the OSLDs effectively integrated dose at all points simultaneously an efficiency noted as an advantage but appeared to over-respond relative to the IC. This over-response can be attributed to several factors: the OSLDs were used without buildup caps (unlike the ion chamber which had a brass buildup), making them more sensitive to lower-energy scattered photons, and their inherent sensitivity and minimal detectable dose on the order of 0.5 mSv could exaggerate readings at the threshold of detection. Similar behavior has been observed in out-of-field dose measurements where OSLDs require careful calibration for the leakage energy spectrum to avoid error on the order of tens of percent. Despite these differences, all three methods in our study identified the same spatial point of maximum leakage, indicating our measurement approach was reproducible and robust. This multi-modal agreement gives confidence that the leakage mapping is accurate. Moreover, our findings align with other modern studies: for instance, Shine et al. used OSLDs to assess a Varian TrueBeam’s head leakage and likewise reported compliance with IEC limits, with extremely low out-of-field photon doses (on the order of 10^− 3^–10^− 2^%) for 10 MV FFF beams^22^. Such concordance with published data reinforces that our measured leakage levels are reliable and that OSLD-based measurements, when properly handled, can serve as a convenient surrogate to ion chambers for simultaneous multi-point leakage detection.

### Leakage in cyberknife S7 vs. earlier models and conventional Linacs

Our investigation confirms that the CyberKnife S7 achieves significantly lower head leakage radiation than earlier CyberKnife models and compares favourably with conventional linear accelerators (LINACs). The evolution of CyberKnife design from the G3/G4 through VSI and M6 to the current S7 model has progressively reduced leakage levels. Early-generation CyberKnife units (G3/G4), which employed basic tungsten shielding and only fixed circular collimators, exhibited leakage often approaching the IEC 60601-2-1 permissible limit of ~ 0.5% of the primary beam^6^. The introduction of the Iris variable-aperture collimator in the CyberKnife VSI brought modest reductions in off-axis leakage through improved collimation, while a major redesign in the M6 model (incorporating the InCise™ multileaf collimator) further tightened shielding and scatter management in the head. Notably, the M6’s multileaf collimator, while improving beam shaping, introduced some leaf transmission; the average leakage through the closed MLC has been reported around 0.22–0.25%, higher than the < 0.12% leakage through closed fixed cones or Iris collimators^23^. Our values were consistent with the manufacturer’s design goal of keeping leakage “consistently below 0.05%” of the primary beam for the S7. When comparing the CyberKnife S7 to conventional LINAC systems, the S7’s performance is equally impressive. Modern gantry-based LINACs (6–10 MV range) typically must also meet the < 0.1% leakage criterion, and published measurements usually find maximal leakage points around 0.1–0.2%. For instance, Taneja et al. reported that a Varian TrueBeam (6 MV) had a worst-case leakage on the order of 0.14% of CAX dose at 1 m (measured at a known “hot spot” on the gantry head), with most other positions an order of magnitude lower^24^. Our CyberKnife S7’s peak leakage percentage is below these values, reflecting the benefit of its dedicated shielding design.

### Clinical and safety implications of low leakage levels

The very low head leakage radiation observed for the CyberKnife S7 carries several positive implications for clinical workflows, shielding design, and patient safety. First and foremost, reduced leakage translates into reduced stray radiation dose to patients and staff. Our results confirm that even at the maximum leakage point, the dose rate escaping the S7 is a minute fraction of the primary beam.

In room shielding design, head leakage is a critical factor in determining secondary barrier requirements. Radiation outside the primary beam (especially at distances beyond ~ 20–30 cm from field edge) is dominated by head leakage rather than patient scatter^24^. Thus, the magnitude of head leakage can directly dictate how thick walls or ceilings need to be for protection. Our finding that S7 leakage is well under the 0.5% IEC recommendation means that the S7 imposes a smaller shielding burden than machines operating closer to the limit. In practical terms, a lower leakage fraction can allow either a reduction in barrier thickness or an increase in workload (patients treated per week) without exceeding regulatory dose limits in uncontrolled areas. Yang et al. showed that in a busy CyberKnife center, the actual workload, use factor, and modulation factor combined were about an order of magnitude lower than the conservative values assumed in shielding guidelines^25^.

For patient safety, minimizing leakage is directly beneficial. While the leakage dose is small per fraction, it contributes to the integral dose received by the patient’s whole body over the course of treatment. Over multiple high-dose stereotactic treatments, even a small reduction in peripheral dose may reduce the already low probability of radiation-induced secondary malignancies or other late effects. In addition to the therapeutic beam, contributions from other radiation sources such as head leakage, patient scatter, and secondary neutrons (for high-energy beams) were also considered, as these components provide important context when evaluating the overall risk profile associated with treatment^26^. By keeping leakage to essentially negligible levels, the CyberKnife S7 helps ensure that patients receive almost no unnecessary radiation outside the target area, an especially reassuring fact for long-term survivorship in younger patients or those receiving repeat radiosurgery courses. Additionally, low head leakage is advantageous for any clinical scenarios that involve concurrent presence of radiation-sensitive devices or implants in the room (for example, certain real-time tumor tracking systems or cardiac monitors), as it reduces the risk of their interference or damage.

### Limitations of the present study and future directions

While this study provided a comprehensive assessment of head leakage using multiple measurement modalities, there are some limitations to acknowledge, which also point toward areas for future improvement. One limitation was the inconsistency in buildup and energy response compensation between detectors. The ionization chamber measurements were performed with a brass buildup cap to establish charged-particle equilibrium, whereas the OSLDs were used in naked form (no additional buildup material) at the measurement points. This means the OSLDs were more sensitive to lower-energy scattered photons and may not have exactly matched the energy response of a tissue-equivalent detector at those points. The lack of buildup could partially explain the higher leakage percentages recorded by OSLDs, as their reading would be inflated by soft X-rays present in head leakage. In future experiments, using water equivalent mini phantoms or buildup caps for all OSLDs would improve the tissue equivalence of the measurements and allow a more apples-to-apples comparison with ion chamber data. Along the same lines, applying an out-of-field calibration factor for OSLDs (accounting for their slight over-response to leakage spectra) would likely reduce the discrepancy with the ion chamber readings^22^.

Another technical limitation was that our measurement used discrete point measurements (nine around the patient plane and additional out-of-plane points as per IEC 60601-2-1). It is possible that very localized “hotspot” areas of leakage (for example, through small seams or ports in the head) could exist that were not exactly co-located with our predefined measurement grid. We mitigated this by observing that all three detector types found the maximum at the same IEC-specified point, suggesting no hidden hotter spot was missed. However, a more fine-grained leakage mapping could be achieved in future studies by techniques such as wrapping the linac head in radiation film or using a two-dimensional detector array. Taneja et al. demonstrated a method of first using radiographic film to identify leakage hotspots on a TrueBeam, then quantifying those with a 2D ion chamber array, which proved efficient and sensitive to small-area leakage features^24^. Adopting a similar approach for the CyberKnife could provide a full leakage radiation distribution and ensure that the absolute maximum leakage location is captured.

Another limitation is related to the dose measurements themselves. Because head leakage radiation is inherently very low, our OSLD readings at some positions were near the minimum detection limit of the dosimeters (on the order of 10^−2 cGy). Although we delivered 500 MU at each orientation (which yielded measurable signals), future studies could improve measurement accuracy by increasing the delivered Mus (e.g., 1000–2000 MU) for leakage tests. This would accumulate a larger dose on passive detectors like OSLDs, improving their signal-to-noise ratio and reducing relative uncertainty (at the cost of slightly longer beam delivery time). We also note that our study was conducted on a CyberKnife S7 system without the InCise MLC installed, using only the fixed cones and Iris collimator. While this allowed us to characterize the leakage for the most common CyberKnife collimation modes, the absence of the MLC means we did not directly measure leakage through the MLC leaves or between them. In an S7 system equipped with the optional MLC, one might expect slightly different leakage characteristics – for instance, leakage through closed MLC leaves might present a distinct distribution (as prior data suggests ~ 0.2% transmission through MLC vs. ~0.1% through fixed apertures)^23^. However, given that the S7’s head was redesigned to accommodate the MLC, it is likely that overall head leakage remains low. Future work should include measurements on an S7 with the MLC to confirm that leakage still meets the stringent criteria and to examine how the MLC leakage pattern (e.g., between leaf ends) contributes to out-of-field dose.

Lastly, it should be mentioned that our use of a pressurized survey meter provided quick real-time readings, but such instruments typically have energy-dependent calibration and angular response that could introduce additional uncertainty. We mitigated this by using the survey meter primarily as a relative indicator and cross-check against the ion chamber. In future investigations, especially if aiming to establish primary standards for leakage dose, one might consider using a large active volume ion chamber specifically designed for leakage measurements or even solid-state detectors with known flat energy response in the leakage range. Additionally, computational modeling (e.g., Monte Carlo simulations of the CyberKnife head) could augment empirical measurements by predicting leakage spectra and distribution. Such simulations, benchmarked against our measured data, would allow exploration of scenarios impractical to measure (for instance, confirming leakage at all gantry angles or evaluating the efficacy of potential shielding upgrades). In summary, by standardizing buildup conditions, employing high sensitivity mapping techniques, and expanding to MLC-equipped systems, future studies can further refine the quantification of head leakage and ensure that next-generation systems push these values even lower.

This study helps fill an important gap in the literature regarding head leakage radiation from advanced robotic radiosurgery systems. To date, relatively few peer-reviewed reports have focused on quantitatively measuring head leakage in CyberKnife units, especially the latest model under clinical conditions. By using three independent dosimetric methods and assessing both in-plane and out-of-plane leakage, we provide a robust dataset characterizing the CyberKnife S7’s leakage profile. These results extend the knowledge from earlier leakage studies on conventional LINACs and older CyberKnife versions into the realm of state-of-the-art SRS technology. Notably, our multi-modal approach demonstrates how different detectors can be employed in a complementary fashion to validate results, an approach that could be adopted as a best-practice during acceptance testing or annual physics QA of high-precision radiotherapy machines. Our work provides a practical methodology and reference data to encourage more widespread verification of head leakage in radiosurgery installations. Ensuring that leakage is within specifications is not merely a regulatory formality, but as we have discussed, it has real implications for shielding design, patient peripheral dose, and long-term safety.

## Conclusion

The head leakage radiation of the CyberKnife System for both in-patient and out-of-patient planes using three different methods: optically stimulated luminescence dosimeters (OSLD), vented ionization chambers (IC), and pressurized ionization chamber-based survey meters (SM). The results demonstrated that the measured leakage levels for both IRIS and FIXED collimator types, in both patient planes, are well within the limits prescribed by the IEC 60601-2-1 standard. Specifically, the in-patient plane leakage was significantly below the 0.1% tolerance, and the out-of-patient plane leakage was well below the 0.5% threshold. This indicates that the CyberKnife System adheres to safety standards, ensuring minimal radiation leakage and safeguarding patient and operator safety.

## Supplementary Information

Below is the link to the electronic supplementary material.

Supplementary Material 1

## Data Availability

All data generated or analysed during this study are included in this published article and its supplementary information files are also shared.
