# Supplementary material for: Quantification of head leakage radiation in CyberKnife robotic radiosurgery systems using a multimodal approach
Source: Sci Rep. 2025 Oct 7;15:34913. doi: 10.1038/s41598-025-18689-1 (PMC12504714; doi:10.1038/s41598-025-18689-1)
Supplement: Supplementary file 1 — Supplementary Material 1 [file 41598_2025_18689_MOESM1_ESM.pdf]

S1: summarizes transmission measurements through a fixed collimator using three dosimetric systems: Ionization Chamber (IC), Survey Meter (SM), and Optically Stimulated Luminescent Dosimeters (OSLD), measurement point (1–16), the table lists fixed collimator readings for all three detectors, followed by their respective transmission values—both as a fraction (unitless) and percentage calculated relative to the open readings. These data reflect the degree of beam attenuation at each point, enabling assessment of the collimator's shielding efficiency across different spatial locations.

	fixed collimator			TRANSMISSION			TRANSMISSION (%)		
	IC(nC)	SM(R/H)	OSLD(mSv)	IC	SM	OSLD			
OPEN READING	113.7	60000	86.8623						
points							IC	SM	OSLD
1	0.023	7.3	0.11858	0.00020	0.00012	0.00137	0.020	0.012	0.137
2	0.02	7.3	0.10581	0.00018	0.00012	0.00122	0.018	0.012	0.122
3	0.024	7.2	0.12273	0.00021	0.00012	0.00141	0.021	0.012	0.141
4	0.021	7.2	0.1122	0.00018	0.00012	0.00129	0.018	0.012	0.129
5	0.0211	7.2	0.12806	0.00019	0.00012	0.00147	0.019	0.012	0.147
6	0.03	7.1	0.12098	0.00026	0.00012	0.00139	0.026	0.012	0.139
7	0.029	7.2	0.14538	0.00026	0.00012	0.00167	0.026	0.012	0.167
8	0.018	7.3	0.09773	0.00016	0.00012	0.00113	0.016	0.012	0.113
10	0.011	5.6	0.05019	0.00010	0.00009	0.00058	0.010	0.009	0.058
11	0.008	3.7	0.04322	0.00007	0.00006	0.00050	0.007	0.006	0.050
12	0.01	5.4	0.04368	0.00009	0.00009	0.00050	0.009	0.009	0.050
13	0.011	6.1	0.04283	0.00010	0.00010	0.00049	0.010	0.010	0.049
14	0.008	4.4	0.04654	0.00007	0.00007	0.00054	0.007	0.007	0.054
15	0.0085	4.2	0.04788	0.00007	0.00007	0.00055	0.007	0.007	0.055
16	0.0098	4.6	0.07176	0.00009	0.00008	0.00083	0.009	0.008	0.083

S2: summarizes transmission measurements through a IRIS collimator using three dosimetric systems: Ionization Chamber (IC), Survey Meter (SM), and Optically Stimulated Luminescent Dosimeters (OSLD), measurement point

	IRIS collimator			TRANSMISSION			TRANSMISSION(%)		
	IC(nC)	SM(R/H)	OSLD(mS	IC	SM	OSLD			
OPEN READING	113.7	60000	86.8623				IC	SM	OSLD
1	0.024	7.2	0.11886	0.0002	0.0001	0.0014	0.021	0.012	0.137
2	0.028	7.2	0.10096	0.0002	0.0001	0.0012	0.025	0.012	0.116
3	0.023	7.2	0.1152	0.0002	0.0001	0.0013	0.020	0.012	0.133
4	0.174	7.2	0.10784	0.0015	0.0001	0.0012	0.153	0.012	0.124
5	0.021	7.2	0.12021	0.0002	0.0001	0.0014	0.018	0.012	0.138
6	0.0257	7.2	0.12775	0.0002	0.0001	0.0015	0.023	0.012	0.147
7	0.0297	7.1	0.14553	0.0003	0.0001	0.0017	0.026	0.012	0.168
8	0.0178	7.3	0.10895	0.0002	0.0001	0.0013	0.016	0.012	0.125
10	0.01	5.4	0.01265	0.0001	0.0001	0.0001	0.009	0.009	0.015
11	0.009	4.3	0.04667	0.0001	0.0001	0.0005	0.008	0.007	0.054
12	0.0094	5.1	0.04498	0.0001	0.0001	0.0005	0.008	0.009	0.052
13	0.0074	5.9	0.04453	0.0001	0.0001	0.0005	0.007	0.010	0.051
14	0.0085	3.7	0.04154	0.0001	0.0001	0.0005	0.007	0.006	0.048
15	0.01	4.9	0.0589	0.0001	0.0001	0.0007	0.009	0.008	0.068
16	0.00927	4.1	0.04703	0.0001	0.0001	0.0005	0.008	0.007	0.054

S3: presents Other than Patient Plane Leakage for both fixed and IRIS collimator. For each collimator type, meter readings (in nC) were recorded at multiple off-axis positions (A to I) using an ionization chamber. The maximum and average transmission percentages are also reported for each configuration, with the fixed collimator showing a maximum of 0.0493% and average of 0.0370%, and the IRIS collimator showing a maximum of 0.0525% and average of 0.0383%, thereby quantifying leakage radiation beyond the treatment field due to collimator scatter or hardware limitations.

	Position	6.4.3 Other than Patient Plane Leakage (FIXED)	
			MU Delivered 500 MU
		Meter Readings in nC	*Percentage of Transmission*
60 mm Collimator		9.727	
Blank Collimator	A	0.0034	0.0350
	B	0.0038	0.0391
	C	0.0043	0.0442
	D	0.0034	0.0350
	E	0.0039	0.0401
	F	0.0048	0.0493
	G	0.0038	0.0391
	H	0.003	0.0308
	I	0.002	0.0206
		Max % of Transmission	0.0493
		Avg % of Transmission	0.0370

	Position	6.4.3 Other than Patient Plane Leakage (IRIS)	
			MU Delivered 500 MU
		Meter Readings in nC	*Percentage of Transmission*
Irish		9.713	
Fully Closed	A	0.002	0.0206
	B	0.003	0.0309
	C	0.0023	0.0237
	D	0.0032	0.0329
	E	0.0043	0.0443
	F	0.0043	0.0443
	G	0.0045	0.0463
	H	0.0051	0.0525
	I	0.0048	0.0494
		Max % of Transmission	0.0525
		Avg % of Transmission	0.0383
